# A Novel Use of Integra™ Bilayer Matrix Wound Dressing on a Pediatric Scalp Avulsion: A Case Report

**Published:** 2015-03-05

**Authors:** Michael Singer, Jessica Korsh, William Predun, Dennis Warfield, Richard Huynh, Thomas Davenport, Louis Riina

**Affiliations:** ^a^Nassau University Medical Center, Garden City, NY; ^b^Long Island Plastic Surgical Group, Garden City, NY

**Keywords:** avulsion, Integra, wound, matrix, alopecia

## Abstract

**Objective:** Soft tissue injuries with full-thickness skin involvement not amenable to local flaps may be treated with dermal matrices and subsequent skin grafting. **Methods:** A pediatric patient presented with a 50-cm^2^ scalp avulsion down to periosteum and outer cranial table post-vehicular trauma. After cultivating healthy cranial neodermis with Integra Bilayer Matrix Wound Dressing, a novel modification of treatment protocol was attempted by removal of the silastic layer. **Results:** Neodermis transformation to granulation tissue followed by contraction of the wound reduced alopecia while also eliminating the need for a split-thickness skin graft to the area. **Conclusion:** A novel modification of treatment protocols utilizing acellular dermal matrices improved aesthetic outcomes and may present a cost-, time-, and procedure-sparing treatment option for avulsion injuries.

In instances when local flaps are not available when treating tissue avulsions, acellular dermal matrices are often used in healing areas of exposed calvarium. Avulsions often necessitate 2-stage reconstruction with dermal matrix and full- or split-thickness skin grafting (STSG). We present the case of a 7-year-old boy struck by an automobile resulting in a 50-cm^2^ scalp avulsion down to periosteum and outer cranial table directly above the outer calvarium. STSG was planned as part of treatment protocol including creation of neodermis. Successful treatment with a novel protocol modification utilizing Integra Bilayer Matrix Wound Dressing (Integra LifeSciences Corporation, Plainsboro, New Jersey) alone negated the necessity for STSG. This case introduces a novel method of treating avulsions with a lower cost of care, less patient morbidity, and more aesthetically pleasing results.

## METHODS

**Figure 1:**
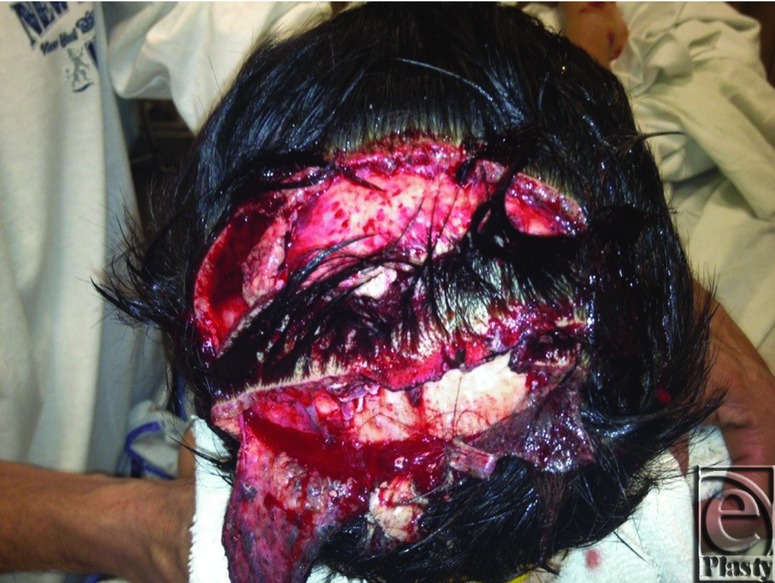
Preoperative care.

**Figure 2:**
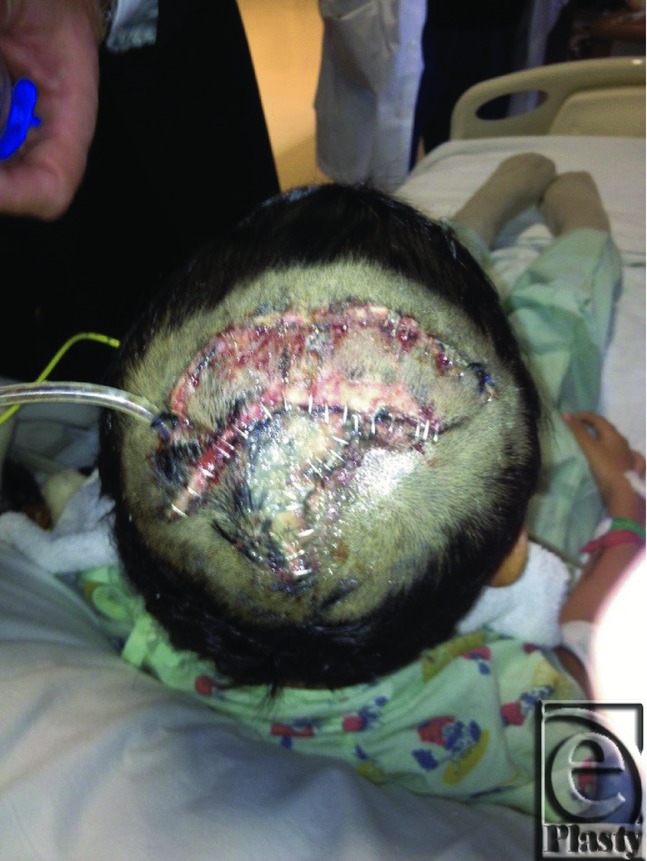
The wound 4 days after the application of Integra.

**Figure 3:**
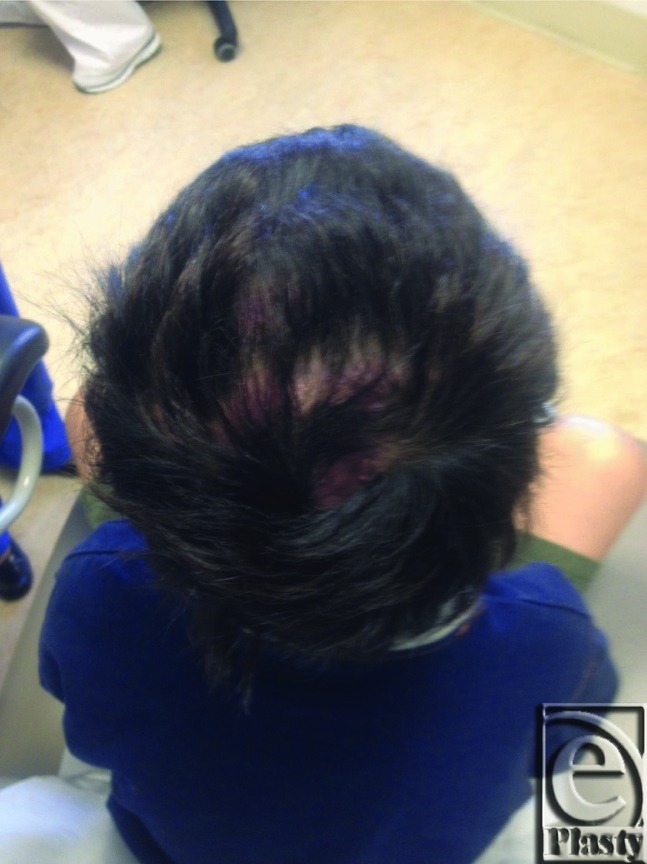
The final result, 170 days after treatment.

A 7-year-old male patient sustained a 50-cm^2^ scalp avulsion down to periosteum and cranial outer table of bone (Fig 1) after being struck by an automobile. Wound debridement preceded layered plastic closure and advancement flap closure to all amenable areas but exposed periosteum and outer cranial table wounds. Because of the large surface area of the wound, the original plan was to close the avulsion with the creation of neodermis using Integra at the site and a delayed STSG to follow. After observing the take of the Integra layer, a decision was made to forego STSG to the neodermis in favor of removing the silastic layer to induce transformation into granulation tissue. The final step utilized silver nitrate (AgNO_3_) modulation of subsequent myofibroblast-mediated contraction (Fig 2) until closure. The wound was closed with a good aesthetic result and no residual alopecia (Fig 3).

## DISCUSSION

Extensive scalp wounds and avulsions with exposed periosteum are treated with skin grafts or left to heal by secondary intention when wounds are smaller. Those wounds with exposed calvarium are typically treated with dermal substitutes due to the extended interval to contracture, infection risk, and difficulty with dressing changes.^1^

In this patient, a novel modification of Integra protocol was applied with optimal aesthetic outcome. Integra was utilized to regenerate neodermis in areas of extensive wound damage as indicated.^2^ We selected Integra because the product traditionally acts as a scaffold allowing for capillary and cellular regeneration to occur in a multifaceted way.^3^ Specifically (*a*) the product's outer semipermeable silicone layer acts as a temporary epidermis, controlling water vapor loss, covering the site of the wound, and protecting the wound from external pathogens, while (*b*) the inner collagen-glycosaminoglycan biodegradable matrix provides a platform for cellular repopulation and capillary regeneration, ultimately allowing the wound to properly close.^3^^,^^4^

Our reported use of Integra is novel in that removal of the silastic layer upon vascularization of the Integra neodermis allowed for transformation of neodermis to granulation tissue.^4^^,^^5^ While the initial treatment plan included a delayed STSG due to the large surface area (50 cm^2^) of the avulsion, observation of rapid wound contracture after removal of the Integra silastic layer rendered a graft unnecessary. This finding is significant for several reasons, including (*a*) minimized alopecia, as STSG may not support hair follicles^6^^,^^7^; (*b*) decreased number of procedures, and associated reductions in length of care and complications or comorbidities associated with additional or staged interventions; (*c*) prevention of STSG-associated hyper- and hypopigmentation of both the graft donor and recipient sites^8^; (*d*) avoidance of STSG-associated risk factors documented to cause graft failure, such as infection, hematoma, and poor adhesion secondary to graft trauma or motion^6^; and (*e*) overall superior aesthetic result.

The efficacy of this approach may also be evaluated following additional cost-benefit analyses of the use of Integra in healing acute traumatic wounds by comparing costs between (*a*) treatment with subsequent STSG and (*b*) treatment via reepithelialization without STSG. No such studies could be identified at the time of publication. However, an analysis was conducted in 2006 to determine the economic benefit of Integra versus STSG in chronic wounds,^9^ citing Integra as an equally economically viable and more aesthetically pleasing alternative. Although promising, the study included nontraumatic chronic wounds and did not control for the initial use of Integra.^9^ However, there is evidence that supports this approach from a cost-effective point of view. Single rather than secondary or staged treatment reduces cost of care and associated risks of complications or comorbidities. STSG requires an additional surgical operation, which—depending on the size—can cost tens of thousands of dollars.^9^ In addition, research shows that STSG exhibits failure rates near 20%.^10^^,^^11^ Finally, avoiding an STSG eliminates the cost associated with dressing changes administered by a visiting nursing service.^9^

This protocol is only useful in limited applications and will not be universally appropriate for avulsion injuries. The authors suggest further research on the efficacy of this treatment to determine (*a*) the successful contracture rate of traumatic wounds post-Integra application (without subsequent skin grafting) and (*b*) whether such treatment displays decreasing rates of successful contracture with increasing wound surface area. The author suggests that practitioners revaluate reconstructive protocols and allow for controlled contracture when it stands to benefit patient function or aesthetic outcomes.

## CONCLUSION

Reepithelialization via the use of Integra has the potential to minimize the occurrence of complications that are characteristic of skin grafts. It also enabled the patient's scalp to heal without noticeable sequelae, in this case alopecia, while eliminating the need for a donor site and preventing an additional visit to the operating room. These prospects, combined with a projected lower cost versus a skin graft (both procedural cost and from foregone morbidities), are promising and call for additional research into the efficacy of our approach.
